# Impact of shared storybook reading on child development: The Japan Environment and Children’s Study

**DOI:** 10.1038/s41390-025-04721-7

**Published:** 2026-01-08

**Authors:** Haruhiko Nakamura, Tomohisa Suzuki, Keita Kanamori, Chiharu Ota, The Japan Environment and Children’s Study Group, Michihiro Kamijima, Shin Yamazaki, Maki Fukami, Reiko Kishi, Koichi Hashimoto, Kenichi Sakurai, Shuichi Ito, Ryoji Shinohara, Hidekuni Inadera, Takeo Nakayama, Ryo Kawasaki, Yasuhiro Takeshima, Hideki Nagashima, Narufumi Suganuma, Mayumi Tsuji, Kimitoshi Nakamura

**Affiliations:** 1 Department of Development and Environmental Medicine, Tohoku University Graduate School of Medicine, Sendai, Japan; 2 Department of Pediatrics, Tohoku University Hospital, Sendai, Japan; 3 Environment and Genome Research Center, Tohoku University Graduate School of Medicine, Sendai, Japan; 4 Principal Investigator, Nagoya City University, Nagoya, Japan; 5 National Institute for Environmental Studies, Tsukuba, Japan; 6 National Center for Child Health and Development, Tokyo, Japan; 7 Hokkaido University, Sapporo, Japan; 8 Fukushima Medical University, Fukushima, Japan; 9 Chiba University, Chiba, Japan; 10 Yokohama City University, Yokohama, Japan; 11 University of Yamanashi, Chuo, Japan; 12 University of Toyama, Toyama, Japan; 13 Kyoto University, Kyoto, Japan; 14 Osaka University, Suita, Japan; 15 Hyogo Medical University, Nishinomiya, Japan; 16 Tottori University, Yonago, Japan; 17 Kochi University, Nankoku, Japan; 18 Kyushu University, Fukuoka, Japan; 19 Kumamoto University, Kumamoto, Japan

## Abstract

**Background:**

Shared storybook reading in early childhood is crucial for cognitive and social development, but its association with broader developmental domains is less established. This study aimed to investigate the association between the frequency of shared storybook reading and overall child development.

**Methods:**

This study analyzed data from the Japan Environment and Children’s Study, a nationwide prospective birth cohort study. The frequency of shared storybook reading was assessed at multiple time points up to age 3. Child development was assessed using the Ages and Stages Questionnaire-3 (ASQ-3). The association was analyzed using multivariate models adjusted for parental background, opportunities to play with the child, and media exposure.

**Results:**

A total of 36,866 mother-child pairs were analyzed. The frequency of storybook reading positively correlated with the ASQ-3 scores across all developmental domains. After adjustments, frequent reading at age 3 was associated with a 5.5-point increase (95% CI: 5.0–6.0, *p* < 0.001) in communication scores compared to rare reading. Among children whose ASQ-3 scores fell below the cutoff at the age of 1 year, more frequent reading at subsequent ages was associated with greater score improvements.

**Conclusion:**

Frequent shared storybook reading positively correlated with the overall developmental progress in early childhood.

**Impact:**

- Based on a large-scale nationwide birth cohort study, frequent shared storybook reading is positively associated with the overall developmental progress in early childhood.
- Even for children with developmental delays at age one, continuous reading was positively associated with their development.

## Introduction

Infancy is a critical period for cognitive, motor, and social development.^1^ A child’s development is influenced by various factors, such as health, nutrition, safety, appropriate caregiving, and early learning.^1^ The longitudinal and bidirectional interactions between the child and the caregiver contribute to favorable child development.^2^ Reading storybooks is primarily for fun. However, it can also foster bidirectional communication between parents and children.^3^ Furthermore, reading storybooks can act as an intervention that provides or enhances psychosocial stimulation, contributing significantly to children’s cognitive and language development.^4^ Previous research has shown that early storybook reading may positively affect children’s language abilities and social behavior.^5–7^ Additionally, reading books together increases the frequency of shared reading activities.^7^

While various aspects of neural and motor development contribute to the acquisition of language skills in early childhood, the association between storybook reading and neural-motor development has not been widely studied.^8^ The Japan Environment and Children’s Study (JECS) is an ongoing nationwide birth cohort study, aiming to investigate environmental factors that may influence children’s health and development.^9^ Through self-administered questionnaires and medical records transcripts, the study collects data on demographic factors, lifestyle, socioeconomic status, environmental exposures, medical history, childbirth information, and developmental assessments using the Ages and Stages Questionnaire, Third Edition (ASQ-3).^9,10^ This study aimed to investigate the relationship between the frequency of shared storybook reading and overall child development across five key developmental domains (communication, gross motor, fine motor, problem-solving, and personal-social behavior) using the ASQ-3. Furthermore, considering that shared reading is part of the broader home life environment, we also explored its association with other factors such as parent-child playtime and children’s media exposure. By analyzing a large Japanese cohort dataset in this study, we aimed to understand the association between shared storybook reading and ASQ-3 scores.

## Method

### Dataset

This study utilized a dataset from the JECS, a nationwide birth cohort study currently ongoing in Japan. Pregnant women were enrolled in the JECS between January 2011 and March 2014. Participants were recruited from 15 Regional Centres across Japan, including Hokkaido, Miyagi, Fukushima, Chiba, Kanagawa, Koshin, Toyama, Aichi, Kyoto, Osaka, Hyogo, Tottori, Kochi, Fukuoka, South Kyushu, and Okinawa. For the present study, we analyzed the dataset (jecs-ta-20190930) released in October 2019. The JECS protocol was reviewed and approved by the Ministry of the Environment’s Institutional Review Board on Epidemiological Studies and the Ethics Committees of all participating institutions (Ethical Number: No.100910001). All procedures and experiments commenced after written informed consent was obtained from the participants. The dataset primarily consisted of caregiver responses to questionnaires. Details of the questionnaires have been described previously. Self-administered questionnaires were answered at several time points: before childbirth (for maternal health and lifestyle) and at 1 year, 1.5 years, 2 years, 2.5 years, and 3 years after childbirth (for child-related information).^9,10^ The specific questionnaire items and response options used to assess the primary exposure (shared storybook reading) and key covariates (including opportunities to play and media exposure) at each time point are detailed in Supplementary Table S1.

### Study participants

Of the 104,062 fetuses originally registered, we excluded the participants with incomplete ASQ-3 outcomes (1, 1.5, 2, 2.5, and 3 years), or missing shared storybook reading data or other covariates (1, 2, and 3 years). No specific exclusion criteria were applied for preterm infants or children with known developmental conditions; all participants with available data were included.

### Outcomes

In this study, the Japanese-translated version of the ASQ-3 was used for developmental assessments. The Japanese version of the ASQ-3 (J-ASQ-3) consists of age-specific questionnaires designed for children between 6 and 60 months.^11^ The questionnaire assesses development in five domains (communication, gross motor, fine motor, problem-solving, and personal-social skills) from infancy through early childhood. Each of the five domains includes six questions, and respondents answer each item by selecting “yes” (10 points), “sometimes” (5 points), or “not yet” (0 points). The total score for each domain is the sum of the points. A total score in each domain falls below -2 standard deviations (SD) from the mean, it suggests developmental delay.^11^ We defined the cutoff as a score below -2 SD from the mean of our study sample at each time point. This approach was chosen over using fixed, previously published cutoffs to better reflect the distribution of developmental scores within our specific large-scale cohort.

### Exposures

Reading frequency at each age was assessed based on the responses to the question, “How often do you talk to your child while showing them a storybook?” There were five response categories: rarely, 1–3 times per month, 1–2 times per week, 3–4 times per week, and 5 or more times per week. For our analysis, we consolidated the five response categories into three groups: “Always” group (comprising respondents who chose ‘5 or more times per week’), ‘Sometimes’ group (comprising the three intermediate categories: ‘1–3 times per month,’ ‘1–2 times per week,’ and ‘3–4 times per week’), and ‘Rarely’ group (comprising respondents who chose ‘rarely’).

### Statistical analyses

To examine the relationship between reading frequency at each age and the ASQ-3 scores at 3 years, we performed a general linear model analysis. First, we conducted a crude analysis that did not adjust for other factors (Model 0). We then examined a model adjusted for factors known from previous literature.^12^ Model 1 was adjusted for maternal and paternal education, maternal smoking during pregnancy, and annual household income. Model 2 was further adjusted for the time spent with the child, such as opportunities to play with the child, daily media exposure, and time spent using a computer or smartphone near the child at each age. Only individuals without missing data were included in the analysis. To examine the longitudinal impact of reading frequency on the ASQ-3 scores, we focused on children who scored below the cutoff point at 1 year of age. These children were categorized into three groups based on their storybook reading frequency over the subsequent three years as follows. 1. ‘Consistently Always’ group: caregivers reported shaded storybook reading to their child more than five times per week at all time points. 2. ‘Consistently Rarely’ group: caregivers reported reading less than once per month across all three assessments. 3. ‘Intermittently Sometimes’ group: children whose reported reading frequency fell between the other two groups.

Furthermore, to compare ASQ scores at 3 years of age among the three groups, t-tests were conducted with Bonferroni correction applied for multiple comparisons. Statistical significance was set at *p* < 0.05. All statistical analyses were conducted using R (ver. 4.4.1) software.

## Results

**Fig. 1:**
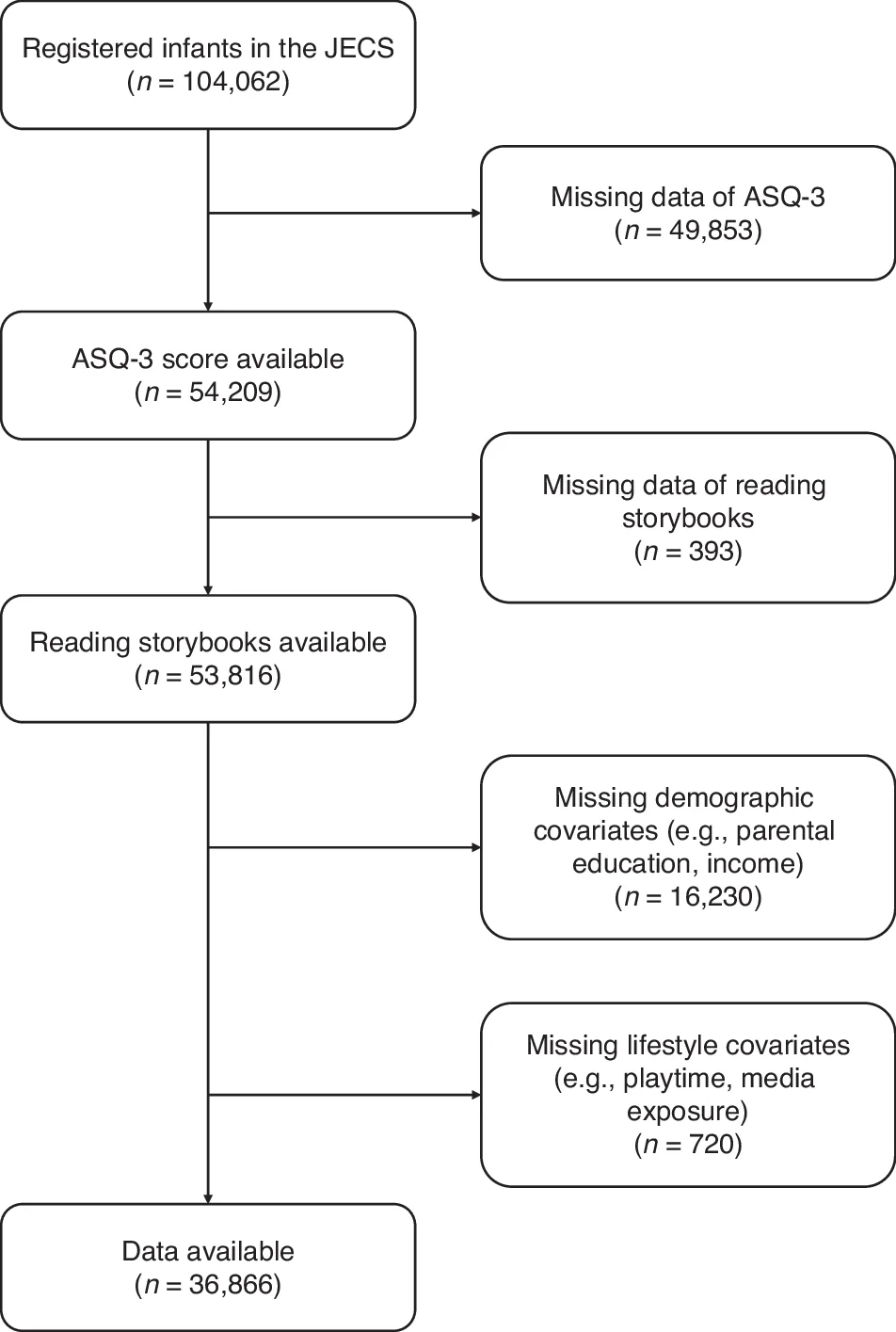
Selection Process for Participants. Flowchart of participants. The first set of covariates (demographic) included maternal and paternal education, maternal smoking during pregnancy, and household annual income. The second set of covariates (lifestyle) included opportunities to play with the child, daily media exposure, and time spent using a computer or smartphone near the child at each age.

Of the 104,062 fetuses originally registered, 67,196 were excluded because of missing ASQ-3 outcomes, shared storybook reading data, or other covariates at 1, 2, and 3 years of age. Consequently, 36,866 participants were included in the final analysis. (Fig. 1)

The frequency of shared storybook reading at 1 year of age and participant characteristics are presented in Table 1. There were 10,942 participants in the ‘always’ group, 22,262 in the ‘sometimes’ group, and 3662 in the ‘rarely’ group, indicating that approximately one-third of participants frequently read to their child. Participant characteristics included covariates, such as maternal BMI, maternal smoking habits, household income, parents’ educational level, and the child’s sex. Moreover, a higher storybook reading frequency was associated with lower maternal smoking rates, higher household income levels, and greater maternal educational attainment.

**Table 1:** Baseline Profiles by Storybook Reading Frequency at Age 1.

	Always	Sometimes	Rarely
	*N* = 10,942^*1*^	*N* = 22,262^*1*^	*N* = 3662^*1*^
Mother’s BMI	20.31 (18.98, 22.03)	20.55 (19.14, 22.46)	20.69 (19.17, 22.67)
Pre-pregnancy alcohol consumption			
Non-drinker	9,831 (90%)	20,394 (92%)	3,419 (93%)
Drinker (grams of pure alcohol)			
Less than 11.5 grams	643 (5.9%)	1,095 (4.9%)	141 (3.9%)
11.6–23 grams	323 (3.0%)	520 (2.3%)	64 (1.7%)
24–46 grams	99 (0.9%)	206 (0.9%)	26 (0.7%)
47–69 grams	29 (0.3%)	26 (0.1%)	9 (0.2%)
70–92 grams of pure alcohol	11 (0.1%)	10 ( <0.1%)	1 ( <0.1%)
93–138 grams of pure alcohol	5 ( <0.1%)	7 ( <0.1%)	1 ( <0.1%)
More	1 ( <0.1%)	4 ( <0.1%)	1 ( <0.1%)
Mother’s smoking behavior			
Never smoked	7093 (65%)	13,268 (60%)	1905 (52%)
Ever smoked			
Used to smoke, but quit before realizing this pregnancy	2473 (23%)	5497 (25%)	1000 (27%)
Used to smoke, but quit after realizing this pregnancy	1174 (11%)	2830 (13%)	561 (15%)
Currently smoking (1 to 10 cigarettes per day)	163 (1.5%)	560 (2.5%)	147 (4.0%)
Currently smoking (11 to 20 cigarettes per day)	38 (0.3%)	104 (0.5%)	48 (1.3%)
Currently smoking (more than 21 cigarettes per day)	1 ( <0.1%)	3 ( <0.1%)	1 ( <0.1%)
Annual income			
Less than 2 million yen	398 (3.6%)	1,016 (4.6%)	251 (6.9%)
2–4 million yen	3306 (30%)	7,702 (35%)	1,358 (37%)
4–6 million yen	3658 (33%)	7640 (34%)	1214 (33%)
6–8 million yen	2061 (19%)	3536 (16%)	507 (14%)
8–10 million yen	910 (8.3%)	1,475 (6.6%)	209 (5.7%)
10–12 million yen	372 (3.4%)	505 (2.3%)	67 (1.8%)
12–15 million yen	130 (1.2%)	208 (0.9%)	26 (0.7%)
15–20 million yen	66 (0.6%)	115 (0.5%)	22 (0.6%)
More than 20 million yen	41 (0.4%)	65 (0.3%)	8 (0.2%)
Mother’s education level			
Junior high school	235 (2.1%)	734 (3.3%)	204 (5.6%)
High school	2495 (23%)	6,731 (30%)	1,403 (38%)
Technical college	163 (1.5%)	354 (1.6%)	59 (1.6%)
Vocational school	2377 (22%)	5315 (24%)	857 (23%)
Junior college	2134 (20%)	4090 (18%)	570 (16%)
University	3,273 (30%)	4,730 (21%)	534 (15%)
Graduate school (Master’s/Doctorate)	265 (2.4%)	308 (1.4%)	35 (1.0%)
Father’s education level			
Junior high school	441 (4.0%)	1,349 (6.1%)	342 (9.3%)
High school	3,201 (29%)	8,171 (37%)	1,501 (41%)
Technical college	208 (1.9%)	468 (2.1%)	80 (2.2%)
Vocational school	2081 (19%)	4255 (19%)	666 (18%)
Junior college	242 (2.2%)	496 (2.2%)	80 (2.2%)
University	4004 (37%)	6577 (30%)	872 (24%)
Graduate school (Master’s/Doctorate)	765 (7.0%)	946 (4.2%)	121 (3.3%)
Child’s gender			
Male	5209 (48%)	11,451 (51%)	2095 (57%)
Female	5733 (52%)	10,811 (49%)	1567 (43%)
Mode of delivery			
Vaginal delivery	5942 (54%)	13,009 (58%)	2179 (60%)
Induced labor	2229 (20%)	3926 (18%)	611 (17%)
Vacuum extraction	758 (6.9%)	1291 (5.8%)	191 (5.2%)
Forceps delivery	38 (0.3%)	43 (0.2%)	6 (0.2%)
Cesarean section	1975 (18%)	3993 (18%)	675 (18%)
Gestational age			
Term (>=37 weeks)	10,825 (99%)	22,008 (99%)	3614 (99%)
Preterm ( <37 weeks)	117 (1.1%)	254 (1.1%)	48 (1.3%)
Birth weight	3030 (2802, 3,276)	3042 (2802, 3290)	3048 (2806, 3290)
Physical abnormalities in the newborn			
None	10,302 (94%)	20,984 (94%)	3451 (94%)
Present	640 (5.8%)	1278 (5.7%)	211 (5.8%)

^*1*^ Median (Q1, Q3); n (%)

^*2*^ For context, the median annual household income in Japan during the study enrollment period (2011-2014) was approximately 4.4 million yen^26^.

Table 2 shows the relationship between the frequency of shared storybook reading each year and the frequency of playing with their child, the amount of time the child spent watching TV or DVDs, and the amount of time spent using a computer or mobile phone near the awake child. At 1 year of age, 98% of the children in the ‘Consistently Always’ group played with their parents five or more times per week, compared to 79% in the ‘Consistently Rarely’ group (*p* < 0.001 for overall association). This pattern was maintained at 2 and 3 years of age, with 85% and 80% of the ‘Consistently Always’ group experiencing daily playtime at 2 and 3 years of age, respectively. Conversely, the ‘Consistently Rarely’ group had consistently lower frequencies (53% at 2 years old.43% at 3 years old). Additionally, children in the ‘Consistently Rarely’ group consistently exhibited longer screen time. At the age of 1 year, 10% of the children in the ‘Consistently Rarely’ group watched TV or DVDs for four or more hours per day, compared to 5.4% in the ‘Consistently Always’ group (*p* < 0.001 for overall association). Although the proportion of children with extended screen time decreased with age in both groups, the difference persisted. At 2 and 3 years of age, 2.7% and 1.4% of the ‘Consistently Rarely’ group continued to have four or more hours of daily screen time, respectively. In contrast, the rates were substantially lower in the ‘Consistently Always group (0.4% at 2 years old and <0.1% at 3 years old). Moreover, parental screen use while the child was awake showed a similar trend. At 1 year of age, only 1.0% of parents in the ‘Consistently Always’ group used digital devices for ≥4 hours/day in the presence of the awake child, compared to 3.1% in the ‘Consistently Rarely’ group (p < 0.001 for overall association). Although this proportion increased slightly in the ‘Consistently Always’ group at 2 (2.6%) and 3 (2.3%) years of age, the rates remained lower than those in the ‘Consistently Rarely’ group (11% at 2 and 6.0% at 3 years of age). Our findings demonstrated that frequent storybook reading was associated with increased child-parent playing time, reduced child media exposure, and decreased parental media usage in the presence of children.

**Table 2:** Parent-Child Interactions and Media Exposure by Storybook Reading Frequency at Each Age.

Age	storybook reading frequency			*p*-value^2^
1year	Always	Sometimes	Rarely	
	*N* = 10,942^*1*^	*N* = 22,262^*1*^	*N* = 3,662^*1*^	
The frequency of playing with their child (1 year)				<0.001
Rarely	5 ( <0.1%)	16 ( <0.1%)	44 (1.2%)	
1 to 3 times a month	17 (0.2%)	83 (0.4%)	33 (0.9%)	
1 to 2 times a week	127 (1.2%)	1,246 (5.6%)	402 (11%)	
3 to 4 times a week	120 (1.1%)	1284 (5.8%)	274 (7.5%)	
5 times or more per week	10,673 (98%)	19,633 (88%)	2909 (79%)	
The amount of time in a day their child spends watching TV or DVDs (1 year)				<0.001
None	1034 (9.4%)	2190 (9.8%)	499 (14%)	
Less than 1 hour	3839 (35%)	7514 (34%)	1,121 (31%)	
1 to 2 hours	3436 (31%)	6659 (30%)	928 (25%)	
2 to 4 hours	2043 (19%)	4481 (20%)	740 (20%)	
4 hours or more	590 (5.4%)	1418 (6.4%)	374 (10%)	
The amount of time spent using a computer or mobile phone (including emailing, gaming, etc.) while your child is awake during the day (1 year)				<0.001
None	1445 (13%)	2461 (11%)	428 (12%)	
Less than 1 hour	6951 (64%)	13,381 (60%)	2,073 (57%)	
1 to 2 hours	1788 (16%)	4329 (19%)	699 (19%)	
2 to 4 hours	645 (5.9%)	1740 (7.8%)	348 (9.5%)	
4 hours or more	113 (1.0%)	351 (1.6%)	114 (3.1%)	
**2 years**	**Always**	**Sometimes**	**Rarely**	
	*N* = 12,352^*1*^	*N* = 22,965^*1*^	*N* = 1,549^*1*^	
The frequency of playing with their child (2 year)				<0.001
Rarely	12 (<0.1%)	34 (0.1%)	43 (2.8%)	
1 to 3 times a month	41 (0.3%)	243 (1.1%)	72 (4.6%)	
1 to 2 times a week	1114 (9.0%)	4611 (20%)	432 (28%)	
3 to 4 times a week	683 (5.5%)	3127 (14%)	178 (11%)	
5 times or more per week	10,502 (85%)	14,950 (65%)	824 (53%)	
The amount of time spent using a computer or mobile phone (including emailing, gaming, etc.) while your child is awake during the day (2 years)				<0.001
None	2504 (20%)	3804 (17%)	224 (14%)	
Less than 1 hour	8009 (65%)	14,395 (63%)	855 (55%)	
1 to 2 hours	1473 (12%)	3697 (16%)	295 (19%)	
2 to 4 hours	318 (2.6%)	938 (4.1%)	133 (8.6%)	
4 hours or more	48 (0.4%)	131 (0.6%)	42 (2.7%)	
The amount of time in a day their child spends watching TV or DVDs (2 year)				<0.001
None	345 (2.8%)	348 (1.5%)	30 (1.9%)	
Less than 1 hour	3532 (29%)	6097 (27%)	316 (20%)	
1 to 2 hours	5242 (42%)	9893 (43%)	566 (37%)	
2 to 4 hours	2793 (23%)	5515 (24%)	462 (30%)	
4 hours or more	440 (3.6%)	1112 (4.8%)	175 (11%)	
**3 years**	**Always**	**Sometimes**	**Rarely**	
	*N* = 10,587^*1*^	*N* = 24,211^*1*^	*N* = 2068^*1*^	
The frequency of playing with their child (3 year)				<0.001
Rarely	14 (0.1%)	61 (0.3%)	74 (3.6%)	
1 to 3 times a month	86 (0.8%)	462 (1.9%)	129 (6.2%)	
1 to 2 times a week	1231 (12%)	5527 (23%)	698 (34%)	
3 to 4 times a week	824 (7.8%)	3753 (16%)	269 (13%)	
5 times or more per week	8,432 (80%)	14,408 (60%)	898 (43%)	
The amount of time your child typically spends interacting with devices such as mobile phones, handheld information terminals, or electronic gaming devices in a day.				<0.001
None	6235 (59%)	11,842 (49%)	910 (44%)	
Less than 1 hour	3803 (36%)	9866 (41%)	707 (34%)	
1 to 2 hours	446 (4.2%)	1965 (8.1%)	288 (14%)	
2 to 4 hours	94 (0.9%)	479 (2.0%)	135 (6.5%)	
4 hours or more	9 (<0.1%)	59 (0.2%)	28 (1.4%)	
The amount of time in a day their child spends watching TV or DVDs (3 years)				<0.001
None	1196 (11%)	2345 (9.7%)	310 (15%)	
Less than 1 hour	5034 (48%)	10,329 (43%)	793 (38%)	
1 to 2 hours	2955 (28%)	7608 (31%)	538 (26%)	
2 to 4 hours	1159 (11%)	3280 (14%)	302 (15%)	
4 hours or more	243 (2.3%)	649 (2.7%)	125 (6.0%)	

^*1*^ *n* (%)

^*2*^ *P*-values were calculated using the Chi-squared test for the association between storybook reading frequency and each variable.

Table 3 shows the association between the frequency of shared storybook reading at 1, 2, and 3 years of age, and ASQ-3 communication scores at 3 years old. In Model 0, the communication scores increased progressively with higher shared storybook reading frequencies. The scores increased with reading frequency, reaching up to 6.3 points higher at 2 years old and 6.4 points higher at 3 years old. In Model 1, the scores also increased, reaching up to 5.5 points higher at 2 years old and 5.6 points higher at 3 years old. In Model 2, the score increase remained significant, reaching up to 5.1 points higher at 2 years old and 5.5 points higher at 3 years old. These results showed that at all ages and under all models of adjustment, a higher frequency of storybook reading and an increase in ASQ-3 communication scores were significantly associated. Interestingly, the scores for the other developmental domains also increased with an increased frequency of storybook reading (Supplementary Table S2).

**Table 3:** Regression Analysis of ASQ Communication Scores at Age 3 and Storybook Reading Frequency at Each Age.

3-year ASQ Communication score	Univariate regression analysis			Multivariable regression analysis					
		model 0			model 1			model 2	
	Beta	95% CI^*1*^	*p*-value	Beta	95% CI^*1*^	*p*-value	Beta	95% CI^*1*^	*p*-value
Frequency of reading story books to the child at 1-year-old.									
Rarely	—	—		—	—		—	—	
1 to 3 times a month	1.6	1.1, 2.1	<0.001	1.4	0.98, 1.9	<0.001	1.4	0.96, 1.9	<0.001
1 to 2 times a week	2.8	2.4, 3.2	<0.001	2.5	2.1, 2.9	<0.001	2.4	2.0, 2.8	<0.001
3 to 4 times a week	3.8	3.4, 4.2	<0.001	3.2	2.8, 3.6	<0.001	3.2	2.7, 3.6	<0.001
5 times or more per week	4.7	4.3, 5.1	<0.001	4	3.6, 4.4	<0.001	3.9	3.5, 4.3	<0.001
Frequency of reading story books to the child at 2-year-old.									
Rarely	—	—		—	—		—	—	
1 to 3 times a month	2.4	1.8, 3.0	<0.001	2.2	1.6, 2.8	<0.001	1.8	1.2, 2.4	<0.001
1 to 2 times a week	4.4	3.8, 5.0	<0.001	3.9	3.4, 4.5	<0.001	3.5	3.0, 4.1	<0.001
3 to 4 times a week	5.7	5.1, 6.3	<0.001	5	4.5, 5.6	<0.001	4.6	4.1, 5.2	<0.001
5 times or more per week	6.3	5.7, 6.8	<0.001	5.5	5.0, 6.0	<0.001	5.1	4.6, 5.7	<0.001
Frequency of reading story books to the child at 3-year-old.									
Rarely	—	—		—	—		—	—	
1 to 3 times a month	3.3	2.7, 3.8	<0.001	3	2.5, 3.5	<0.001	2.8	2.3, 3.3	<0.001
1 to 2 times a week	4.5	4.0, 5.0	<0.001	4	3.5, 4.5	<0.001	3.8	3.4, 4.3	<0.001
3 to 4 times a week	5.4	4.9, 5.9	<0.001	4.7	4.2, 5.2	<0.001	4.6	4.1, 5.1	<0.001
5 times or more per week	6.4	5.9, 6.9	<0.001	5.6	5.1, 6.1	<0.001	5.5	5.0, 6.0	<0.001

^*1*^ *CI* Confidence Interval

Model 0: Crude model. Model 1: Adjusted for maternal and paternal education, maternal smoking during pregnancy, and household annual income. Model 2: Additionally adjusted for time spent with the child, such as opportunities to play with the child, daily media exposure, and time spent using a computer or smartphone near the child at each age.

**Fig. 2:**
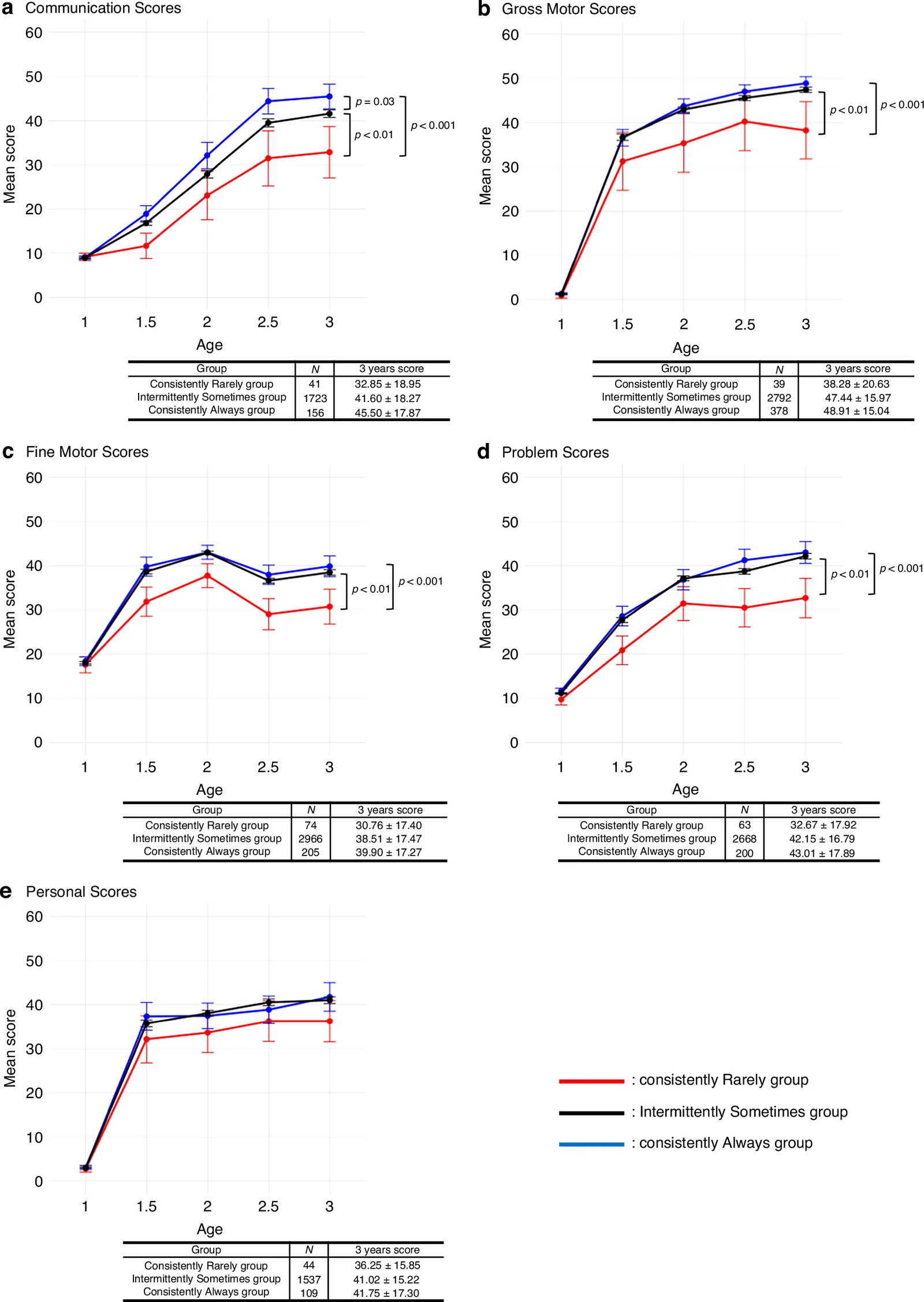
Trends in ASQ-3 Scores Categorized by Consistent Storybook Reading Frequency. A bar chart illustrates the progression of ASQ-3 scores (**a** communication, **b** gross motor, **c** fine motor, **d** problem-solving, and **e** personal-social behavior) over time among children who scored below the cutoff at 1 year of age, stratified by the frequency of storybook reading. Participants were categorized into three groups: ‘Consistently Always’, ‘Intermittently Sometimes’, and ‘Consistently Rarely’. The table shows the number of participants and the unadjusted mean ASQ-3 scores at age 3, presented as mean ± standard deviation.

Next, we examined the developmental promotional effects of establishing a storybook reading routine specifically for children who scored below the ASQ-3 cutoff at 1 year of age. As shown in Fig. 2, the unadjusted mean scores in all developmental domains were highest in the ‘Consistently Always’ group, followed by the ‘Intermittently Sometimes’ group, and lowest in the ‘Consistently Rarely’ group. Statistically significant differences were observed between the ‘Consistently Rarely’ group and the other two groups in all four domains, except for the personal-social domain. In the communication domain, a significant difference was found between the ‘Consistently Always’ and ‘Intermittently Sometimes’ groups.

## Discussion

This study investigated the relationship between the frequency of shared storybook reading and broader developmental outcomes in infants in the first 3 years of life. The results demonstrated a significant association between frequent storybook reading at each age and higher scores in the five developmental domains (communication, gross motor, fine motor, problem-solving, and personal-social behavior) as assessed by the ASQ-3.

While several studies have explored the relationship between storybook reading, language abilities, and social behaviors,^5–7^ their associations with developmental outcomes remain under-researched. This study specifically examined the relationship at 3 years of age, as assessed by the ASQ-3. Our results revealed a significant association between the frequency of storybook reading at each age and higher ASQ-3 scores across all five developmental domains. Importantly, this association remained significant even after adjusting for potential confounders such as the opportunities to play with the child and media exposure, suggesting an independent positive correlation of shared storybook reading. Moreover, maintaining a high frequency of storybook reading over the first 3 years was found to be associated with overall improved ASQ-3 scores among children with developmental delays at the age of one.

Storybook reading is associated with the acquisition of literacy skills during early childhood. The development of literacy skills requires the establishment of a reading network that integrates vision, language, attention, and executive functions.^13,14^ Early exposure to reading contributes to the construction of this network. Therefore, it is reasonable to conclude that storybook reading enhances communication and social skills during the development process.

Additionally, storybook reading has the potential to simulate joint attention,^15^ which is a critical developmental milestone in infancy. Since joint attention may play a key role in enhancing social skills, storybook reading contributes positively to social development. Additionally, the interactive nature of reading books, which encompasses activities, such as asking questions, gestures, and facial expressions between parents and children, may play a role in fostering neurodevelopmental growth and enhancing social skills.

Notably, this study demonstrated a negative correlation between the duration of storybook reading and both the child’s screen time and the parent’s screen usage in the child’s presence. Similarly, increased storybook reading has been reported to reduce screen time.^16,17^ Importantly, screen time negatively impacts child development.^18,19^ Increasing the frequency of storybook reading has the potential to mitigate the adverse effects associated with screen time.

This study revealed the relationship between storybook reading and motor development. Previous studies have reported a connection between language ability and gross motor skills,^20^ suggesting that posture during activities such as reading storybooks may also promote motor development.^21^ The increased movement involved in reaching for the hands may also stimulate fine motor skill development. Although this study did not evaluate posture while reading, it is possible that the physical stance adopted during such activities indirectly contributes to the developmental progress.

Japan has a high literacy rate and advanced reading comprehension skills relative to other countries at 15 years of age.^22^ In Japan, the *Act on Promotion of Children’s Reading* was enacted in 2001, initiating efforts to encourage reading activities among children.^23^ Around the same time, the implementation of “morning reading” programs targeting students from elementary to high school became widespread within the school education system, potentially contributing to the development of advanced reading comprehension skills. Moreover, engaging in storybook reading during the preschool years is considered essential for bridging early childhood development with formal school education.

Globally, initiatives to enhance the frequency of reading among preschool-aged children are being actively promoted. Healthy People 2030 has established a target to increase the proportion of families reading to children under the age of five years at least four days per week, with the goal of reaching 63.2%.^24^ In our study, the proportion of parents reading to their children three or more times per week at age three years was approximately 50%, indicating that there is considerable potential for further promotion of storybook reading practices. Furthermore, integrating storybook reading into daily routines from an early age can significantly increase its frequency.^25^ Promoting storybook reading from early childhood could further support the establishment of lifelong reading habits, potentially enhancing future cognitive and developmental outcomes.

### Limitations

Although this large-scale study had notable strengths, several limitations must be acknowledged. First, the data were based on self-reports from parents, which may introduce bias or inaccuracies in reporting the frequency of shared storybook reading or the developmental progress of their children. Second, our analysis required complete data across all five ASQ-3 domains at all time points. This resulted in a large number of participants being excluded due to missing data (n = 49,853, with missing data at each time point: 24,841 (23.9%) at 1 year, 29,403 (28.3%) at 1.5 years, 25,250 (24.3%) at 2 years, 27,407 (26.3%) at 2.5 years, and 26,006 (25.0%) at 3 years), and therefore, selection bias may exist. Third, our questionnaire assessed the frequency (how often) of shared reading, but not its duration (how long) or quality (e.g., interactive style). Although we adjusted for the frequency of parent-child play, we could not fully analyze the time allocation between reading and other activities or completely disentangle their independent effects. Fourth, because this was a cross-sectional study, causality cannot be established. Although there is a strong association between shared storybook reading and developmental outcomes, we cannot definitively conclude that shared storybook reading has directly led to these improvements. Longitudinal studies are needed to confirm the long-term effects of shared storybook reading on children’s development.

## Conclusion

In this study, we used a large-scale cohort dataset from Japan to examine the correlation between the frequency of shared storybook reading and early childhood development. The results demonstrated a positive correlation between reading frequency and all ASQ-3 developmental assessment domains, with the scores increasing further with continuous reading habits. However, the causality remains unclear. Further research is needed to fully elucidate how shared storybook reading promotes early childhood development.

## Supplementary information

Supplementary tables

## Data Availability

Data are unsuitable for public deposition due to ethical restrictions and legal framework of Japan. It is prohibited by the Act on the Protection of Personal Information (Act No. 57 of 30 May 2003, amendment on 9 September 2015) to publicly deposit the data containing personal information. Ethical Guidelines for Medical and Health Research Involving Human Subjects enforced by the Japan Ministry of Education, Culture, Sports, Science and Technology and the Ministry of Health, Labour and Welfare also restricts the open sharing of the epidemiologic data. All inquiries about access to data should be sent to: jecs-en@nies.go.jp. The person responsible for handling enquiries sent to this e-mail address is Dr Shoji F. Nakayama, JECS Programme Office, National Institute for Environmental Studies.
