# Supplementary material for: Impact of shared storybook reading on child development: The Japan Environment and Children’s Study
Source: Pediatr Res. 2026 Jan 8;100(3):1082–90. doi: 10.1038/s41390-025-04721-7 (PMC13672088; doi:10.1038/s41390-025-04721-7)
Supplement: Supplementary file 1 [file 41390_2025_4721_MOESM1_ESM.pdf]

Supplemental Table S1: Questionnaire Items for Key Variables at Ages 1, 2, and 3 Years

Age	Question	Answer
1 year	How often do you have opportunities to play with your child?	1=Rarely, 2=1–3 times per month, 3=1–2 times per week, 4=3–4 times per week, 5=5 or more times per week
	How often do you talk to your child while showing them a storybook?	1=Rarely, 2=1–3 times per month, 3=1–2 times per week, 4=3–4 times per week, 5=5 or more times per week
	On a typical day, about how many hours do you let your child watch TV or DVDs?	1=None, 2=Less than 1 hour, 3=1 to less than 2 hours, 4=2 to less than 4 hours, 5=4 or more hours
	On a typical day, while your child is awake, about how many hours do you spend operating a computer or mobile phone (including email, games, etc.) near them?	1=None, 2=Less than 1 hour, 3=1 to less than 2 hours, 4=2 to less than 4 hours, 5=4 or more hours
2 year	How often do you have opportunities to play with your child?	1=Rarely, 2=1–3 times per month, 3=1–2 times per week, 4=3–4 times per week, 5=5 or more times per week
	How often do you talk to your child while showing them a storybook?	1=Rarely, 2=1–3 times per month, 3=1–2 times per week, 4=3–4 times per week, 5=5 or more times per week
	On a typical day, while your child is awake, about how many hours do you spend operating a computer or mobile phone (including email, games, etc.) near them?	1=None, 2=Less than 1 hour, 3=1 to less than 2 hours, 4=2 to less than 4 hours, 5=4 or more hours
	On a typical day, about how many hours do you let your child watch TV or DVDs?	1=None, 2=Less than 1 hour, 3=1 to less than 2 hours, 4=2 to less than 4 hours 5=4 or more hours
3 year	How often do you have opportunities to play with your child?	1=Rarely, 2=1–3 times per month, 3=1–2 times per week, 4=3–4 times per week, 5=5 or more times per week
	How often do you talk to your child while showing them a storybook?	1=Rarely, 2=1–3 times per month, 3=1–2 times per week, 4=3–4 times per week, 5=5 or more times per week
	On a typical day, about how many hours do you watch TV or DVDs with your child?	1=None, 2=Less than 1 hour, 3=1 to less than 2 hours, 4=2 to less than 4 hours, 5=4 or more hours
	On a typical day, about how many hours does your child touch or play with devices such as mobile phones, handheld information terminals, or electronic gaming devices?	1=None, 2=Less than 1 hour, 3=1 to less than 2 hours, 4=2 to less than 4 hours, 5=4 or more hours

Supplemental Table S2: Regression Analysis of ASQ Scores at Age 3 and Shared Storybook Reading Frequency at Each Age

**3-year ASQ Gross Motor score**	**Univariate regression analysis**			**Multivariable regression analysis**					
	**model 0**			**model 1**			**model 2**		
	**Beta**	**95% CI**[1]	**p-value**	**Beta**	**95% CI**[1]	**p-value**	**Beta**	**95% CI**[1]	**p-value**
Frequency of reading story books to the child at 1 year old									
Rarely	—	—		—	—		—	—	
1 to 3 times a month	0.95	0.58, 1.3	<0.001	0.97	0.61, 1.3	<0.001	0.93	0.56, 1.3	<0.001
1 to 2 times a week	1.1	0.76, 1.4	<0.001	1.1	0.75, 1.4	<0.001	0.97	0.65, 1.3	<0.001
3 to 4 times a week	1.5	1.2, 1.8	<0.001	1.5	1.2, 1.8	<0.001	1.4	1.0, 1.7	<0.001
5 times or more per week	1.7	1.4, 2.0	<0.001	1.7	1.4, 2.1	<0.001	1.6	1.3, 1.9	<0.001
Frequency of reading story books to the child at 2 year old									
Rarely	—	—		—	—		—	—	
1 to 3 times a month	0.68	0.20, 1.2	0.006	0.7	0.21, 1.2	0.004	0.47	-0.03, 0.95	0.065
1 to 2 times a week	1.2	0.72, 1.6	<0.001	1.2	0.73, 1.6	<0.001	0.84	0.40, 1.3	<0.001
3 to 4 times a week	1.6	1.1, 2.0	<0.001	1.6	1.1, 2.0	<0.001	1.2	0.75, 1.7	<0.001
5 times or more per week	1.6	1.1, 2.0	<0.001	1.6	1.2, 2.1	<0.001	1.2	0.75, 1.6	<0.001
Frequency of reading story books to the child at 3 year old									
Rarely	—	—		—	—		—	—	
1 to 3 times a month	0.77	0.35, 1.2	<0.001	0.76	0.34, 1.2	<0.001	0.66	0.23, 1.1	0.002
1 to 2 times a week	1.3	0.87, 1.6	<0.001	1.3	0.89, 1.7	<0.001	1.1	0.76, 1.5	<0.001
3 to 4 times a week	1.5	1.1, 1.9	<0.001	1.6	1.2, 2.0	<0.001	1.4	1.0, 1.8	<0.001
5 times or more per week	1.6	1.2, 2.0	<0.001	1.7	1.3, 2.1	<0.001	1.6	1.2, 2.0	<0.001

3-year ASQ Fine Motor score	Univariate regression analysis			Multivariable regression analysis					
	model 0			model 1			model 2		
	**Beta**	**95% CI[1]**	**p-value**	**Beta**	**95% CI[1]**	**p-value**	**Beta**	**95% CI[1]**	**p-value**
Frequency of reading story books to the child at 1 year old									
Rarely	—	—		—	—		—	—	
1 to 3 times a month	2	1.4, 2.5	<0.001	1.8	1.2, 2.3	<0.001	1.7	1.2, 2.3	<0.001
1 to 2 times a week	3.2	2.8, 3.7	<0.001	2.8	2.4, 3.3	<0.001	2.7	2.3, 3.2	<0.001
3 to 4 times a week	4.5	4.1, 5.0	<0.001	3.9	3.4, 4.4	<0.001	3.3	3.3, 4.2	<0.001
5 times or more per week	5.4	5.0, 5.9	<0.001	4.6	4.2, 5.1	<0.001	4.4	3.9, 4.8	<0.001
Frequency of reading story books to the child at 2 year old									
Rarely	—	—		—	—		—	—	
1 to 3 times a month	2.6	1.9, 3.4	<0.001	2.4	1.6, 3.1	<0.001	2	1.3, 2.8	<0.001
1 to 2 times a week	4.2	3.5, 4.9	<0.001	3.7	3.0, 4.3	<0.001	3.1	2.5, 3.8	<0.001
3 to 4 times a week	5.4	4.8, 6.1	<0.001	4.7	4.0, 5.3	<0.001	4	3.3, 4.7	<0.001
5 times or more per week	6.3	5.6, 6.9	<0.001	5.4	4.8, 6.0	<0.001	4.6	4.0, 5.3	<0.001
Frequency of reading story books to the child at 3 year old									
Rarely	—	—		—	—		—	—	
1 to 3 times a month	2.8	2.1, 3.4	<0.001	2.4	1.8, 3.1	<0.001	2.2	1.5, 2.8	<0.001
1 to 2 times a week	4.1	3.5, 4.7	<0.001	3.7	3.0, 4.1	<0.001	3.2	2.6, 3.7	<0.001
3 to 4 times a week	5.2	4.6, 5.8	<0.001	4.4	3.8, 5.0	<0.001	4	3.4, 4.6	<0.001
5 times or more per week	6.3	5.7, 6.9	<0.001	5.4	4.8, 5.9	<0.001	4.8	4.2, 5.4	<0.001

3-year ASQ Problem score	Univariate regression analysis			Multivariable regression analysis					
	model 0			model 1			model 2		
	Beta	95% CI[1]	p-value	Beta	95% CI[1]	p-value	Beta	95% CI[1]	p-value
Frequency of reading story books to the child at 1 year old									
Rarely	—	—		—	—		—	—	
1 to 3 times a month	1.6	1.1, 2.1	<0.001	1.5	1.0, 2.0	<0.001	1.5	0.98, 1.9	<0.001
1 to 2 times a week	2.6	2.2, 3.0	<0.001	2.4	2.0, 2.8	<0.001	2.3	1.9, 2.7	<0.001
3 to 4 times a week	3.5	3.1, 3.9	<0.001	3.1	2.7, 3.5	<0.001	3	2.6, 3.4	<0.001
5 times or more per week	4.3	3.9, 4.7	<0.001	3.8	3.4, 4.2	<0.001	3.7	3.3, 4.1	<0.001
Frequency of reading story books to the child at 2 year old									
Rarely	—	—		—	—		—	—	
1 to 3 times a month	2.4	1.8, 3.0	<0.001	2.3	1.6, 2.9	<0.001	2	1.4, 2.6	<0.001
1 to 2 times a week	4.1	3.5, 4.6	<0.001	3.8	3.2, 4.3	<0.001	3.4	2.8, 3.9	<0.001
3 to 4 times a week	5	4.4, 5.6	<0.001	4.6	4.0, 5.2	<0.001	4.2	3.6, 4.7	<0.001
5 times or more per week	5.6	5.0, 6.1	<0.001	5.1	4.5, 5.7	<0.001	4.6	4.1, 5.2	<0.001
Frequency of reading story books to the child at 3 year old									
Rarely	—	—		—	—		—	—	
1 to 3 times a month	2.8	2.3, 3.4	<0.001	2.7	2.1, 3.2	<0.001	2.4	1.9, 3.0	<0.001
1 to 2 times a week	3.6	3.1, 4.1	<0.001	3.3	2.8, 3.8	<0.001	3	2.5, 3.6	<0.001
3 to 4 times a week	4.6	4.1, 5.2	<0.001	4.2	3.7, 4.7	<0.001	3.9	3.4, 4.4	<0.001
5 times or more per week	5.4	4.9, 6.0	<0.001	4.9	4.4, 5.5	<0.001	4.6	4.1, 5.2	<0.001

3-year ASQ Personal score	Univariate regression analysis			Multivariable regression analysis					
	model 0			model 1			model 2		
	Beta	95% CI[1]	p-value	Beta	95% CI[1]	p-value	Beta	95% CI[1]	p-value
Frequency of reading story books to the child at 1 year old									
Rarely	—	—		—	—		—	—	
1 to 3 times a month	1.5	1.1, 2.0	<0.001	1.3	0.90, 1.8	<0.001	1.3	0.89, 1.7	<0.001
1 to 2 times a week	2.5	2.1, 2.8	<0.001	2.1	1.8, 2.5	<0.001	2.1	1.7, 2.4	<0.001
3 to 4 times a week	3.2	2.8, 3.6	<0.001	2.7	2.3, 3.1	<0.001	2.6	2.3, 3.0	<0.001
5 times or more per week	3.6	3.2, 4.0	<0.001	3	2.6, 3.4	<0.001	2.9	2.6, 3.3	<0.001
Frequency of reading story books to the child at 2 year old									
Rarely	—	—		—	—		—	—	
1 to 3 times a month	2.2	1.6, 2.8	<0.001	2	1.4, 2.6	<0.001	1.6	1.1, 2.2	<0.001
1 to 2 times a week	3.6	3.1, 4.2	<0.001	3.2	2.7, 3.7	<0.001	2.7	2.2, 3.2	<0.001
3 to 4 times a week	4.5	4.0, 5.0	<0.001	3.9	3.4, 4.4	<0.001	3.5	3.0, 4.0	<0.001
5 times or more per week	4.5	4.0, 5.0	<0.001	3.9	3.4, 4.4	<0.001	3.5	3.0, 4.0	<0.001
Frequency of reading story books to the child at 3 year old									
Rarely	—	—		—	—		—	—	
1 to 3 times a month	2.7	2.2, 3.2	<0.001	2.4	1.9, 2.9	<0.001	2.2	1.7, 2.7	<0.001
1 to 2 times a week	3.5	3.0, 4.0	<0.001	3.1	2.6, 3.5	<0.001	2.9	2.4, 3.4	<0.001
3 to 4 times a week	4.2	3.7, 4.7	<0.001	3.6	3.1, 4.1	<0.001	3.5	3.1, 4.0	<0.001
5 times or more per week	4.5	4.1, 5.0	<0.001	3.9	3.4, 4.3	<0.001	3.9	3.4, 4.3	<0.001

[1] CI = Confidence Interval
